# β-Nb_9_VO_25_

**DOI:** 10.1107/S1600536814007831

**Published:** 2014-04-16

**Authors:** Rawia Nasri, Saïda Fatma Chérif, Mohamed Faouzi Zid, Ahmed Driss

**Affiliations:** a Laboratoire de Matériaux et Cristallochimie, Faculté des Sciences de Tunis, Université de Tunis El Manar, 2092 El Manar Tunis, Tunisia

## Abstract

The title compound, nona­niobium vanadium penta­cosa­oxide, was prepared by a solid-state reaction at 1198 K. It is isotypic with Nb_9_AsO_25_, Nb_9_PO_25_ and Ta_9_VO_25_. The structure consists of NbO_6_ octa­hedra (one with 4/*m*.. and two with *m*.. symmetry) and VO_4_ tetra­hedra (-4.. symmetry) sharing corners and edges to form a three-dimensional framework. This framework can be considered as a junction between ribbons made up from NbO_6_ octa­hedra and chains of NbO_6_ octa­hedra and chains of VO_4_ tetra­hedra. The V site shows half-occupancy, hence one half of the VO_4_ tetra­hedra is unoccupied. The structural differences with α-Nb_9_VO_25_, VOSO_4_, SbOPO_4_ and NbOPO_4_ oxides are discussed.

## Related literature   

For isotypic compouds, see: Ulutagay *et al.* (1998 ▶); Roth *et al.* (1965 ▶); Casais *et al.* (1993 ▶). For physical properties, see: Prabaharan *et al.* (1997 ▶); Aranda *et al.* (1992 ▶); Bergner *et al.* (2009 ▶). For details of structurally related compounds, see: Haddad & Jouini (1996 ▶, 1997 ▶); Zid *et al.* (1992 ▶); Casais *et al.* (1993 ▶); Chérif *et al.* (2011 ▶); Roth *et al.* (1965 ▶); Köhler *et al.* (1989 ▶); Piffard *et al.* (1986 ▶); Longo & Arnott (1970 ▶); Tachez *et al.* (1981 ▶); Amos *et al.* (1998 ▶). For details of bond-valence calculations, see: Brown & Altermatt (1985 ▶).

## Experimental   

### 

#### Crystal data  

Nb_9_VO_25_
*M* *_r_* = 1287.13
Tetragonal, 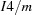

*a* = 15.7726 (9) Å
*c* = 3.8399 (6) Å
*V* = 955.27 (17) Å^3^
*Z* = 2
Mo *K*α radiation
μ = 5.78 mm^−1^
*T* = 298 K
0.14 × 0.05 × 0.04 mm

#### Data collection  

Enraf–Nonius CAD-4 diffractometer
Absorption correction: ψ scan (North *et al.*, 1968 ▶) *T*_min_ = 0.695, *T*_max_ = 0.812
851 measured reflections
593 independent reflections
363 reflections with *I* > 2σ(*I*)
*R*_int_ = 0.044
2 standard reflections every 120 min intensity decay: 1.1%

#### Refinement  

*R*[*F* ^2^ > 2σ(*F* ^2^)] = 0.034
*wR*(*F* ^2^) = 0.086
*S* = 0.99
593 reflections
56 parameters
Δρ_max_ = 1.15 e Å^−3^
Δρ_min_ = −1.52 e Å^−3^

### 

Data collection: *CAD-4 EXPRESS* (Duisenberg, 1992 ▶; Macíček & Yordanov, 1992 ▶); cell refinement: *CAD-4 EXPRESS*; data reduction: *XCAD4* (Harms & Wocadlo, 1995 ▶); program(s) used to solve structure: *SHELXS97* (Sheldrick, 2008 ▶); program(s) used to refine structure: *SHELXL97* (Sheldrick, 2008 ▶); molecular graphics: *DIAMOND* (Brandenburg & Putz, 1999 ▶); software used to prepare material for publication: *WinGX* (Farrugia, 2012 ▶).

## Supplementary Material

Crystal structure: contains datablock(s) I. DOI: 10.1107/S1600536814007831/vn2081sup1.cif

Structure factors: contains datablock(s) I. DOI: 10.1107/S1600536814007831/vn2081Isup2.hkl

CCDC reference: 996072

Additional supporting information:  crystallographic information; 3D view; checkCIF report

## Figures and Tables

**Fig. 1.**
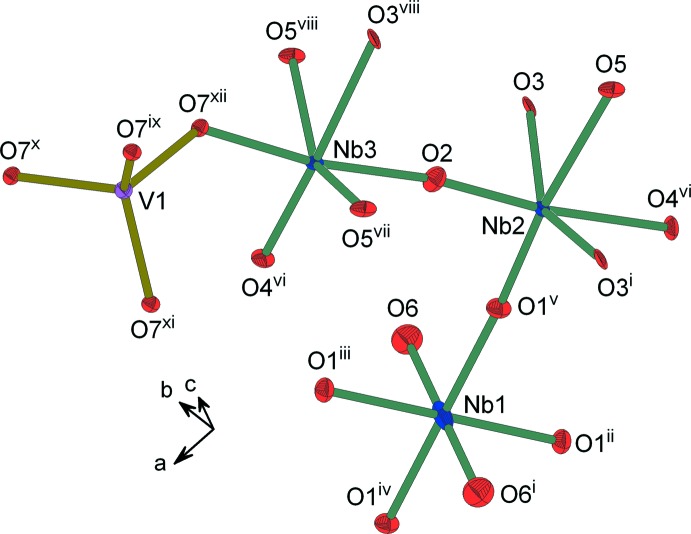
Unité asymétrique dans Nb9VO25. Les éllipsoïdes ont été définis avec 50% de probabilité. [Code de symétrie: (i) x, y, z - 1; (ii)-y, x - 1, z; (iii) y, -x + 1, -z; (iv) x - 1, -y + 1/2, -z - 1/2; (v) -x + 1, -y, -z; (vi) y + 1, -x + 1, -z; (vii) -x + 3/2, -y, z; (viii) -x + 3/2, -y + 1/2, -z + 1/2; (ix) -x + 1, -y + 1, -z + 1; (x) -y + 1/2, x - 1/2, z + 1/2; (xi) y - 1/2, -x + 3/2, -z + 1/2; (xii) x - 1, y, z + 1].

**Fig. 2.**
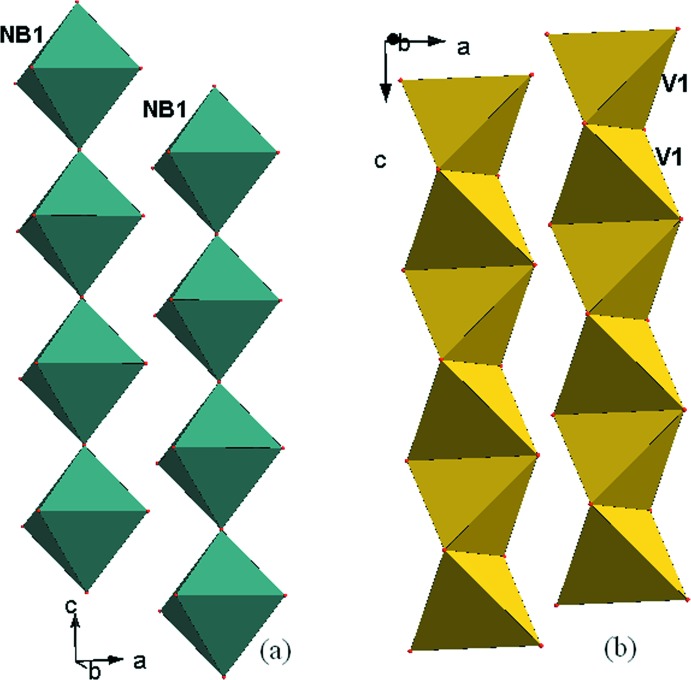
Représentation: (a) des chaînes octaédriques Nb1O5, (b) des chaînes tétraédriques VO2.

**Fig. 3.**
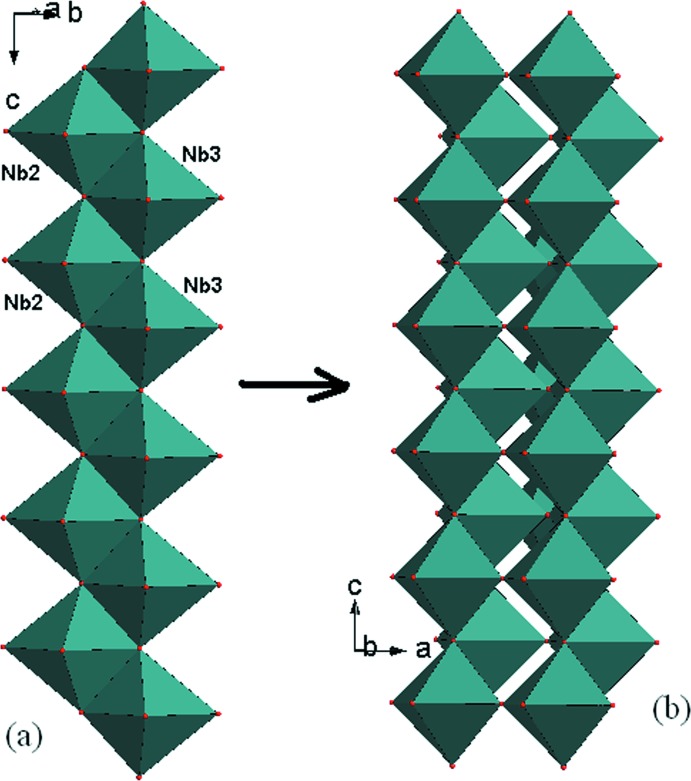
Représentation: (a) des chaînes doubles, (b) des rubans.

**Fig. 4.**
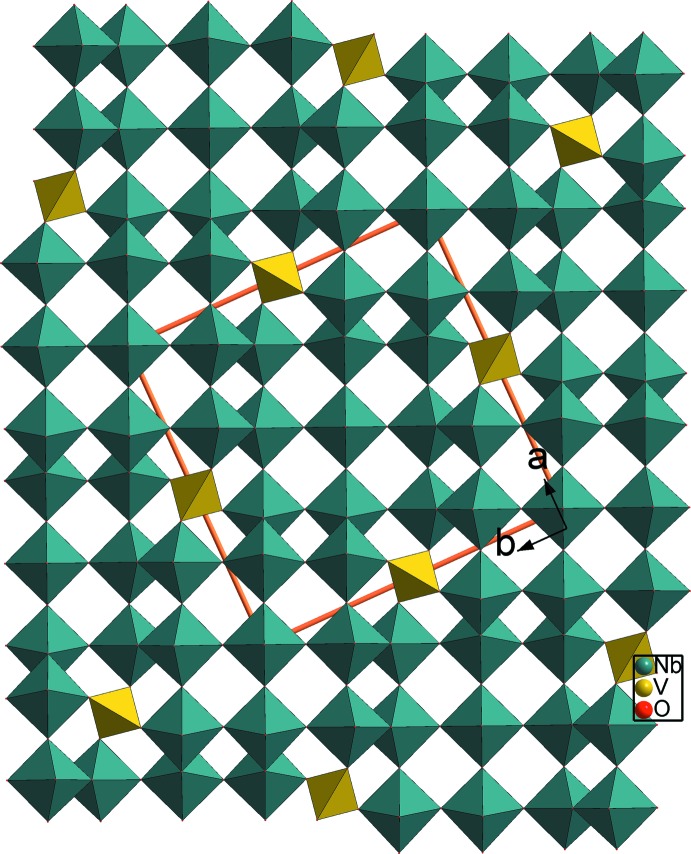
Projection de la structure de β-Nb9VO25 selon [001].

**Fig. 5.**
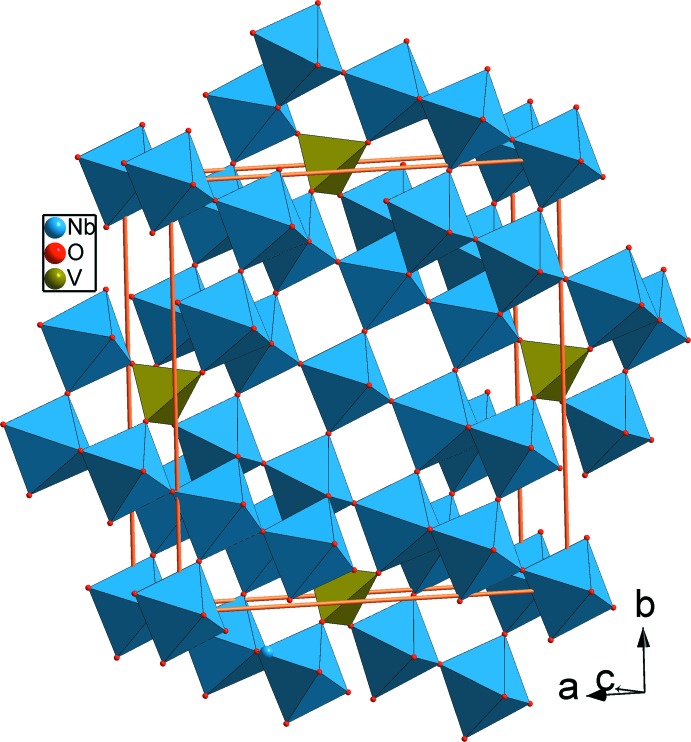
Vue en perspective de la structure de α-Nb9VO25 (Casais et al., 1993) montrant la jonction des polyèdres.

**Fig. 6.**
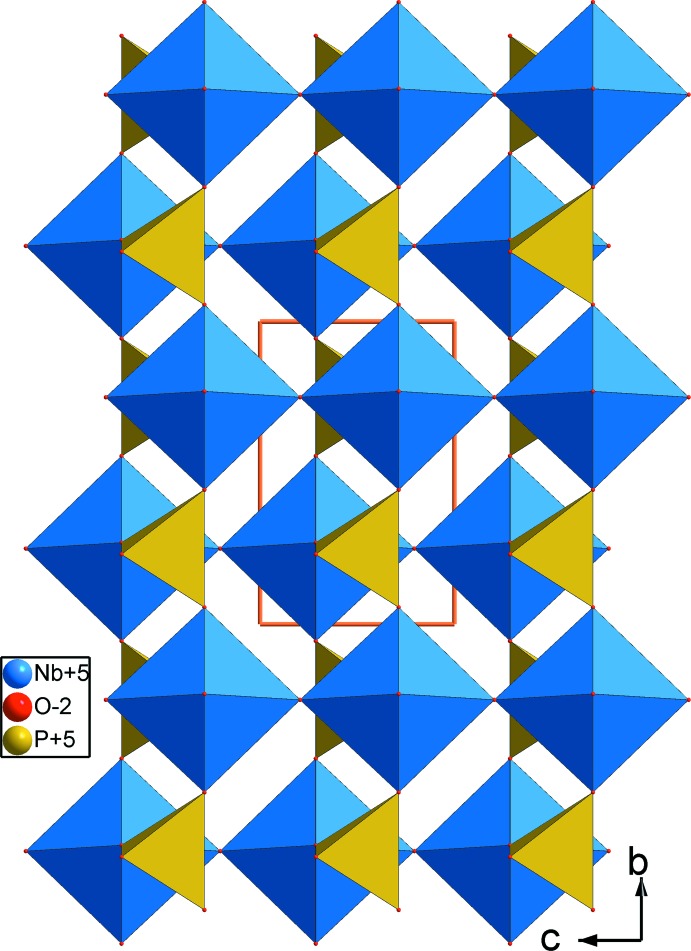
Projection, selon [100], de la structure de NbOPO4 (Amos et al., 1998) montrant la disposition des polyèdres.

## supplementary crystallographic information

### 1. Comment

Les composés à structure ouverte formés d'octaèdres et de tétraèdres constituent un vaste domaine de recherche ces derniéres années. En relation avec leurs structures, ces matériaux présentent des propriétés intéressantes tels que la conductivité ionique (Prabaharan *et al.*, 1997), échange d'ions (Aranda *et al.*, 1992), ou bien elles sont utilisés en catalyse hétérogène (Bergner *et al.*, 2009). Dans ce contexte, nous avons tenté d'explorer les systèmes *A*–Nb–V–O (*A* = cation monovalent) par réaction à l'état solide. L'investigation de ces derniers a permis d'isoler les formes suivantes: NaNb_6.15_V_0.94_O_14_ (Koehler *et al.*, 1989), K_3_Nb_6_VO_19_ (Haddad & Jouini, 1996), Rb_5_VONb_14_O_38_ (Haddad & Jouini, 1997).

Un cristal de forme allongé a été choisi sous microscope polarisant, et s'est averé après étude structurale d'être le composé binaire Nb_9_VO_25_. L'unité asymétrique est constituée par un arrangement de trois octaèdres NbO_6_ et d'un tétraèdre VO_4_ liés entre eux au moyen de sommets, formant ainsi le groupement Nb_3_VO_19_ (Fig. 1). Dans la charpente oxygénée, les octaèdres Nb1O_6_ forment par partage de sommets des chaînes de type Nb1O_5_ (Fig. 2a) et les tétraèdres VO_4_ forment par mise en commun d'arêtes des chaînes de type VO_2_ (Fig. 2b). Par contre, les octaèdres Nb2O_6_ et Nb3O_6_ se lient d'une part par mise en commun de sommets pour former des chaînes classiques NbO_5_ et d'autre part ces dernières se regroupent par partage d'arêtes pour conduire à des chaînes doubles (Fig. 3a), qui par formation de ponts simples Nb2—O—Nb3, et en se regroupant par paires mènent à des rubans (Fig. 3 b). La jonction de ces derniers par mise en commun de sommets avec les chaînes formées par les octaèdres Nb1O_6_ conduit à une charpente octaédrique. La charpente tridimensionnelle de Nb_9_VO_25_ est donc la conséquence d'une jonction entre ces rubans et les chaînes octaédriques Nb1O_5_ et tétraédriques VO_2_ (Fig. 4). Les atomes de niobium et de vanadium forment avec les atomes d'oxygène des liaisons Nb—O et V—O conformes à celles rencontrées dans la littérature (Zid *et al.*, 1992; Casais *et al.*, 1993; Haddad & Jouini, 1996; Chérif *et al.*, 2011).

Le calcul des différentes valences des liaisons (BVS), utilisant la formule empirique de Brown (Brown & Altermatt, 1985), conduit aux valeurs des charges des ions: Nb1(4.944), Nb2(4.897), Nb3(4.928), V1(5.074), en bon accord avec les degrés d'oxydation attendus.

La comparaison de notre structure avec celles des oxydes analogues, montre qu'elle est isotype à: Nb_9_PO_25_ (Roth *et al.*, 1965), Ta_9_VO_25_ (Casais *et al.*, 1993) et Nb_9_AsO_25_ (Ulutagay *et al.*, 1998). Le composé étudié Nb_9_VO_25_ (forme *β*) cristallise dans le système quadratique, groupe d'espace centrosymétrique *I*4/m (87) alors que celui isoformulaire Nb_9_VO_25_ (forme *α*) est non centrosymétrique, groupe d'espace *I*4 (82) (Casais *et al.*, 1993). Une diffèrence nette dans la charpente a été observée. En effet, pour notre composé les tétraèdres se lient par partage d'arêtes, mais pour celui non-centrosymétrique les tétraèdres ne sont pas liés entre eux (Fig. 5). Un examen bibliographique nous a conduit à la famille des oxydes suivants: VOSO_4_ (Longo & Arnott, 1970) (forme *α*) VOPO_4_ (Tachez *et al.*, 1981) SbOPO_4_ (Piffard *et al.*, 1986) et NbOPO_4_ (Amos *et al.*, 1998). Ces derniers présentent une diffèrence nette en mode de connection entre les octaèdres et les tétraèdres. En effet, dans les oxydes SbOPO_4_, NbOPO_4_ et VOSO_4_ les chaînes octaédriques MO_5_ (*M*=Sb, Nb, V) se connectent au moyen de sommets avec les tétraèdres XO_4_ (P, S) pour conduire à une charpente oxygénée tridimensionnelle (Fig. 6) différente à celle rencontrée dans l'oxyde obtenu Nb_9_VO_25_.

### 2. Experimental

Les cristaux de Nb_9_VO_25_ ont été obtenus par réaction à l'état solide à partir des réactifs suivants: Nb_2_O_5_ (FLUKA, 72520), V_2_O_5_ (ACROS ORGANICS, A018448201) et Na_2_CO_3_ (PROLABO, 27778) pris dans les proportions Na:Nb:V=3:6:1. Le mélange, finement broyé, a été mis dans un creuset en porcelaine, placé dans un four puis préchauffé à l'air à 623 K pendant 24 heures en vue d'éliminer les composés volatils: NH_3_ et CO_2_. Il est ensuite porté à une température proche de sa fusion, 1198 K. Le mélange est maintenu à cette température pendant une semaine pour favoriser la germination et la croissance des cristaux puis il subit en premier lieu un refroidissement lent (5°/jour) jusqu'à 1098 K puis un second rapide (50°/h) jusqu'à la température ambiante. Des cristaux de couleur jaune sous forme de baguettes ont été isolés à l'aide d'une loupe binoculaire.

### 3. Refinement

L'analyse de la carte de Fourier différence finale ne révèle aucun pic résiduel significatif. Par ailleurs les ellipsoïdes sont très bien définis. Les densités d'électrons maximum et minimum restants dans la Fourier-différence sont situées respectivements à 0.36 Å de Nb1 et à 1.24 Å de O5. Il en résulte la composition chimique finale, Nb_9_VO_25_ du matériau obtenu.

### Crystal data

**Table:**

Nb_9_VO_25_	*D*_x_ = 4.475 Mg m^−^^3^
*M**_r_* = 1287.13	Mo *K*α radiation, λ = 0.71073 Å
Tetragonal, *I*4/*m*	Cell parameters from 25 reflections
Hall symbol: -I 4	θ = 11–15°
*a* = 15.7726 (9) Å	µ = 5.78 mm^−^^1^
*c* = 3.8399 (6) Å	*T* = 298 K
*V* = 955.27 (17) Å^3^	Prism, yellow
*Z* = 2	0.14 × 0.05 × 0.04 mm
*F*(000) = 1184	

### Data collection

**Table:**

Enraf–Nonius CAD-4 diffractometer	363 reflections with *I* > 2σ(*I*)
Radiation source: fine-focus sealed tube	*R*_int_ = 0.044
Graphite monochromator	θ_max_ = 26.9°, θ_min_ = 2.6°
ω/2θ scans	*h* = −20→1
Absorption correction: ψ scan (North *et al.*, 1968)	*k* = −20→1
*T*_min_ = 0.695, *T*_max_ = 0.812	*l* = −4→1
851 measured reflections	2 standard reflections every 120 min
593 independent reflections	intensity decay: 1.1%

### Refinement

**Table:**

Refinement on *F*^2^	Primary atom site location: structure-invariant direct methods
Least-squares matrix: full	Secondary atom site location: difference Fourier map
*R*[*F*^2^ > 2σ(*F*^2^)] = 0.034	*w* = 1/[σ^2^(*F*_o_^2^) + (0.0234*P*)^2^] where *P* = (*F*_o_^2^ + 2*F*_c_^2^)/3
*wR*(*F*^2^) = 0.086	(Δ/σ)_max_ < 0.001
*S* = 0.99	Δρ_max_ = 1.15 e Å^−^^3^
593 reflections	Δρ_min_ = −1.52 e Å^−^^3^
56 parameters	Extinction correction: *SHELXL97* (Sheldrick, 2008), Fc^*^=kFc[1+0.001xFc^2^λ^3^/sin(2θ)]^-1/4^
0 restraints	Extinction coefficient: 0.0050 (3)

### Special details

**Table:**

Experimental. Le cristal étant de faible taille, la correction d'absorption par psi-scan n'a pas amélioré le résultat de l'affinement.
Geometry. All e.s.d.'s (except the e.s.d. in the dihedral angle between two l.s. planes) are estimated using the full covariance matrix. The cell e.s.d.'s are taken into account individually in the estimation of e.s.d.'s in distances, angles and torsion angles; correlations between e.s.d.'s in cell parameters are only used when they are defined by crystal symmetry. An approximate (isotropic) treatment of cell e.s.d.'s is used for estimating e.s.d.'s involving l.s. planes.
Refinement. Refinement of *F*^2^ against ALL reflections. The weighted *R*-factor *wR* and goodness of fit *S* are based on *F*^2^, conventional *R*-factors *R* are based on *F*, with *F* set to zero for negative *F*^2^. The threshold expression of *F*^2^ > σ(*F*^2^) is used only for calculating *R*-factors(gt) *etc*. and is not relevant to the choice of reflections for refinement. *R*-factors based on *F*^2^ are statistically about twice as large as those based on *F*, and *R*- factors based on ALL data will be even larger.

### Fractional atomic coordinates and isotropic or equivalent isotropic displacement parameters (Å^2^)

**Table:**

	*x*	*y*	*z*	*U*_iso_*/*U*_eq_	Occ. (<1)
Nb1	0.0000	0.0000	0.0000	0.0195 (7)	
Nb2	0.78149 (7)	0.10593 (7)	0.0000	0.0058 (4)	
Nb3	0.88301 (6)	0.32601 (6)	0.0000	0.0057 (3)	
V1	0.0000	0.5000	0.7500	0.0071 (12)	0.50
O1	0.8838 (5)	0.0515 (5)	0.0000	0.012 (2)	
O2	0.8233 (5)	0.2169 (5)	0.0000	0.013 (2)	
O3	0.7479 (5)	0.1109 (5)	0.5000	0.0084 (19)	
O4	0.7185 (5)	−0.0125 (5)	0.0000	0.0106 (19)	
O5	0.6482 (5)	0.1572 (5)	0.0000	0.010 (2)	
O6	0.0000	0.0000	0.5000	0.020 (5)	
O7	0.9251 (5)	0.4473 (5)	0.0000	0.0069 (18)	

### Atomic displacement parameters (Å^2^)

**Table:**

	*U*^11^	*U*^22^	*U*^33^	*U*^12^	*U*^13^	*U*^23^
Nb1	0.0071 (8)	0.0071 (8)	0.044 (2)	0.000	0.000	0.000
Nb2	0.0044 (5)	0.0083 (6)	0.0048 (7)	−0.0002 (4)	0.000	0.000
Nb3	0.0058 (6)	0.0068 (6)	0.0045 (6)	0.0015 (5)	0.000	0.000
V1	0.0072 (17)	0.0072 (17)	0.007 (3)	0.000	0.000	0.000
O1	0.010 (4)	0.012 (4)	0.012 (5)	0.003 (3)	0.000	0.000
O2	0.018 (4)	0.010 (4)	0.012 (5)	−0.002 (4)	0.000	0.000
O3	0.003 (4)	0.011 (4)	0.012 (5)	−0.003 (4)	0.000	0.000
O4	0.009 (4)	0.007 (4)	0.015 (5)	−0.004 (4)	0.000	0.000
O5	0.010 (4)	0.015 (5)	0.004 (5)	0.005 (4)	0.000	0.000
O6	0.023 (7)	0.023 (7)	0.016 (11)	0.000	0.000	0.000
O7	0.008 (4)	0.007 (4)	0.006 (4)	0.000 (3)	0.000	0.000

### Geometric parameters (Å, º)

**Table:**

Nb1—O6^i^	1.9200 (3)	Nb3—O7	2.025 (7)
Nb1—O6	1.9200 (3)	Nb3—O3^viii^	2.292 (8)
Nb1—O1^ii^	2.005 (8)	V1—O7^ix^	1.735 (6)
Nb1—O1^iii^	2.005 (8)	V1—O7^x^	1.735 (6)
Nb1—O1^iv^	2.005 (8)	V1—O7^xi^	1.735 (6)
Nb1—O1^v^	2.005 (8)	V1—O7^xii^	1.735 (6)
Nb2—O1	1.827 (8)	O1—Nb1^xiii^	2.005 (8)
Nb2—O2	1.870 (8)	O3—Nb2^xiv^	1.993 (2)
Nb2—O3^i^	1.993 (2)	O3—Nb3^viii^	2.292 (8)
Nb2—O3	1.993 (2)	O4—Nb3^xv^	1.792 (7)
Nb2—O4	2.115 (7)	O5—Nb3^vii^	1.999 (2)
Nb2—O5	2.253 (8)	O5—Nb3^viii^	1.999 (2)
Nb3—O4^vi^	1.792 (7)	O6—Nb1^xiv^	1.9200 (3)
Nb3—O2	1.962 (8)	O7—V1^ix^	1.735 (6)
Nb3—O5^vii^	1.999 (2)	O7—V1^xvi^	1.735 (6)
Nb3—O5^viii^	1.999 (2)		
			
O6^i^—Nb1—O6	180.0	O2—Nb3—O7	170.5 (3)
O6^i^—Nb1—O1^ii^	90.0	O5^vii^—Nb3—O7	87.5 (2)
O6—Nb1—O1^ii^	90.0	O5^viii^—Nb3—O7	87.5 (2)
O6^i^—Nb1—O1^iii^	90.0	O4^vi^—Nb3—O3^viii^	177.3 (3)
O6—Nb1—O1^iii^	90.0	O2—Nb3—O3^viii^	87.1 (3)
O1^ii^—Nb1—O1^iii^	180.0 (5)	O5^vii^—Nb3—O3^viii^	73.8 (2)
O6^i^—Nb1—O1^iv^	90.0	O5^viii^—Nb3—O3^viii^	73.8 (2)
O6—Nb1—O1^iv^	90.0	O7—Nb3—O3^viii^	83.4 (3)
O1^ii^—Nb1—O1^iv^	90.0	O7^ix^—V1—O7^x^	107.83 (13)
O1^iii^—Nb1—O1^iv^	90.0	O7^ix^—V1—O7^xi^	107.83 (13)
O6^i^—Nb1—O1^v^	90.0	O7^x^—V1—O7^xi^	112.8 (3)
O6—Nb1—O1^v^	90.0	O7^ix^—V1—O7^xii^	112.8 (3)
O1^ii^—Nb1—O1^v^	90.0	O7^x^—V1—O7^xii^	107.83 (13)
O1^iii^—Nb1—O1^v^	90.0	O7^xi^—V1—O7^xii^	107.83 (13)
O1^iv^—Nb1—O1^v^	180.0	O7^ix^—V1—V1^xvii^	123.60 (13)
O1—Nb2—O2	97.3 (3)	O7^x^—V1—V1^xvii^	56.40 (13)
O1—Nb2—O3^i^	104.7 (2)	O7^xi^—V1—V1^xvii^	56.40 (13)
O2—Nb2—O3^i^	93.3 (2)	O7^xii^—V1—V1^xvii^	123.60 (13)
O1—Nb2—O3	104.7 (2)	O7^ix^—V1—V1^xviii^	56.40 (13)
O2—Nb2—O3	93.3 (2)	O7^x^—V1—V1^xviii^	123.60 (13)
O3^i^—Nb2—O3	148.8 (4)	O7^xi^—V1—V1^xviii^	123.60 (13)
O1—Nb2—O4	90.0 (3)	O7^xii^—V1—V1^xviii^	56.40 (13)
O2—Nb2—O4	172.7 (3)	V1^xvii^—V1—V1^xviii^	180.0
O3^i^—Nb2—O4	84.8 (2)	Nb2—O1—Nb1^xiii^	175.9 (5)
O3—Nb2—O4	84.8 (2)	Nb2—O2—Nb3	172.0 (5)
O1—Nb2—O5	173.0 (3)	Nb2^xiv^—O3—Nb2	148.8 (4)
O2—Nb2—O5	89.6 (3)	Nb2^xiv^—O3—Nb3^viii^	104.9 (2)
O3^i^—Nb2—O5	74.8 (2)	Nb2—O3—Nb3^viii^	104.9 (2)
O3—Nb2—O5	74.8 (2)	Nb3^xv^—O4—Nb2	175.1 (5)
O4—Nb2—O5	83.1 (3)	Nb3^vii^—O5—Nb3^viii^	147.6 (4)
O4^vi^—Nb3—O2	95.6 (3)	Nb3^vii^—O5—Nb2	106.1 (2)
O4^vi^—Nb3—O5^vii^	106.1 (2)	Nb3^viii^—O5—Nb2	106.1 (2)
O2—Nb3—O5^vii^	89.9 (2)	Nb1^xiv^—O6—Nb1	180.0
O4^vi^—Nb3—O5^viii^	106.1 (2)	V1^ix^—O7—V1^xvi^	67.2 (3)
O2—Nb3—O5^viii^	89.9 (2)	V1^ix^—O7—Nb3	132.5 (3)
O5^vii^—Nb3—O5^viii^	147.6 (4)	V1^xvi^—O7—Nb3	132.5 (3)
O4^vi^—Nb3—O7	94.0 (3)		

Symmetry codes: (i) *x*, *y*, *z*−1; (ii) −*y*, *x*−1, *z*; (iii) *y*, −*x*+1, −*z*; (iv) *x*−1, *y*, *z*; (v) −*x*+1, −*y*, −*z*; (vi) *y*+1, −*x*+1, −*z*; (vii) −*x*+3/2, −*y*+1/2, −*z*−1/2; (viii) −*x*+3/2, −*y*+1/2, −*z*+1/2; (ix) −*x*+1, −*y*+1, −*z*+1; (x) −*y*+1/2, *x*−1/2, *z*+1/2; (xi) *y*−1/2, −*x*+3/2, −*z*+1/2; (xii) *x*−1, *y*, *z*+1; (xiii) *x*+1, *y*, *z*; (xiv) *x*, *y*, *z*+1; (xv) −*y*+1, *x*−1, *z*; (xvi) *x*+1, *y*, *z*−1; (xvii) −*x*, −*y*+1, −*z*+1; (xviii) −*x*, −*y*+1, −*z*+2.
